# College Notes

**Published:** 1894-01

**Authors:** 


### College Notes.

---

The election of class officers of the class of '94 in the Medical Department of the University of Buffalo was lately held with the following result: President, Myron A. Fisher; Vice-President, Herriott C. Rooth; Secretary, Mrs. Jane North; Treasurer, La Verne C. Colegrove; Orator, Ernest R. Ruffner; Historian, Milton P. Messinger; Prophet, Eugene Caswell; Poet, Miss Angeline D. Smith; Marshal, John Chalmers; Sentinel, Charles F. Tucker.

After the election the successful candidates entertained the class at supper.

